# Levofolene modulates apoptosis induced by 5-fluorouracil through autophagy inhibition: Clinical and occupational implications

**DOI:** 10.3892/ijo.2015.2904

**Published:** 2015-02-24

**Authors:** MONICA LAMBERTI, STEFANIA PORTO, SILVIA ZAPPAVIGNA, PAOLA STIUSO, VIRGINIA TIRINO, VINCENZO DESIDERIO, LUIGI MELE, MICHELE CARAGLIA

**Affiliations:** 1 Department of Experimental Medicine, Section of Occupational Medicine, Second University of Naples, Naples, Italy; 2 Department of Biochemistry, Biophysics and General Pathology, Second University of Naples, Naples, Italy; 3 Section of Biotechnology and Medical Histology, Second University of Naples, Naples, Italy

**Keywords:** levofolene, 5-fluorouracil, apoptosis, keratinocytes, cytotoxicity

## Abstract

5-Fluorouracil (5-FU), often used in combination with levofolene (LF), can induce, as an important side effect, the hand-foot syndrome (HFS) due to toxicity on keratinocytes. This can also damage workers involved in its handling. In the present study, we investigated the mechanisms of the toxicity induced by 5-FU alone or together with LF on human keratinocytes in culture. We found that the two drugs, as expected, had potentiating activity on keratinocyte growth inhibition and that this effect was mediated by induction of apoptosis. In our experimental model, an increased autophagic vacuole accumulation was observed in keratinocytes treated with 5-FU as a significant increase of the monodansylcadaverine (MDC) labeling (marker of late autophagy vacuoles) was recorded. However, the synergism of 5-FU with LF on apoptotic occurrence was not paralleled by a similar increase in autophagic vacuoles at 72 h suggesting an antagonistic effect of LF on autophagy elicited by 5-FU. Differential effects on reactive oxygen species (ROS) elevation in cells treated with 5-FU alone or the combination between 5-FU and LF were also observed. 5-FU induced a time-dependent increase of both O^2−^ and lipid peroxidation while the combination of 5-FU and LF caused a stronger intracellular O^2−^ increase only at 24 h while at 48 and 72 h its effect was lower when compared with that one of 5-FU alone. On the other hand, the addition of LF to 5-FU caused a stronger increase of lipid peroxidation at 48 and 72 h, but its effects were significantly lower at 24 h. These results suggest for the first time that LF potentiates the cytotoxicity of 5-FU on keratinocytes likely through the antagonism on autophagy escape pathway and consequent apoptosis potentiation.

## Introduction

More than six decades after its synthesis (1), 5-fluorouracil (5-FU) remains a key agent, particularly for the treatment of adenocarcinomas of the gastrointestinal tract (2,3). In the United States, preferences for 5-FU administration have gradually shifted from bolus i.v. injection to infusion via a pump to improve safety profile of the drug, because like most chemotherapeutic agents, 5-FU also has numerous toxic effects such as diarrhea, mucositis, myelosuppression and thrombophlebitis of peripheral veins (4). However, more recently, orally delivered derivatives and pro-drugs have been developed. Capecitabine in particular is an oral pro-drug that is ultimately converted to 5-FU by thymidine phosphorylase and uridine phosphorylase, both enzymes are highly expressed in solid tumors allowing for a locally increased concentration of the active drug at the cancer site (5).

Although these systems of drug administration reduces most of the side effects of the therapy, it increases the occurrence of a toxic reaction involving keratinocytes of the palmar region of hands and foot, also called palmar-plantar erythrodysaesthesia or hand-foot syndrome (HFS) (6,7). The symptoms of HFS include numbness, dysaesthesia/paraesthesia, tingling, erythema, painless swelling or discomfort and, in more severe cases, blisters, ulceration, desquamation or severe pain on the hands palms and/or feet soles.

The cellular damage has also been identified due to direct contact of keratinocytes with 5-FU. Therefore, toxic reactions are also possible in healthcare workers, during manipulation stages of antiblastic drugs. Several scientific studies have shown that there is the possibility of exposure to antineoplastic drugs such as doxorubicin, epirubicin, cyclophosphamide and 5-FU in workers (8,9). 5-FU, in particular, has been reported as one of the most concentrated chemotherapeutic drugs in the working areas (950 ng/cm^2^) outside the laminar flow hood, where operators do not use protective gloves (8). 5-FU is often co-administered together with L-folinic acid or levofolene (LF) for their positive interaction on cancer cell growth inhibition due to the formation of a stable ternary complex with 5-FU and tymidilate synthase, the target of 5-FU (10). However, studies are required in order to understand the possible interaction between the two drugs also on the induction of detrimental effects.

In the present study, we studied the effects of LF on the toxicity determined by 5-FU in a model of the human keratinocyte HaCaT cell line. Moreover, we studied the interaction between the two drugs on the cell death mechanisms and oxidative stress in the same *in vitro* model of keratinocytes.

## Materials and methods

### Materials

DMEM, FBS (fetal bovine serum) and tissue culture plastic ware were purchased from Microtech (Naples, Italy). Dihydroethidium (DHE) and monodansylcadaverine (MDC) were purchased from Sigma-Aldrich (Milan, Italy). Annexin V-FITC Apoptosis Detection kit was purchased from eBioscience (San Diego, CA, USA). 5-Fluorouracil (5-FU), doxorubicin (DOXO) and Levofolene (LF) were a gift of Dr Gaetano Facchini (I.N.T. ‘Pascale’, Naples, Italy).

### Cell culture and cell viability assay

The human keratinocyte HaCaT cell line was obtained from American Type Tissue Culture Collection (Rockville, MD) and grown in DMEM supplemented with 10% heat-inactivated fetal bovine serum, 20 mM HEPES, 100 U/ml penicillin, 100 mg/ml streptomycin, 1% L-glutamine and 1% sodium pyruvate. Cells were cultured at 37°C in a 5% CO_2_-95% air environment in humidified incubator. Proliferation of HaCaT cells was performed in the presence of 5-FU with or without 10^−4^ M of LF and DOXO. After trypsinization, cells were plated in 100 μl of medium in 96-well plates at a density of 6×10^3^ per well. Cells were treated 24 h later with increasing concentrations of pharmacological agents ranging from 6–2.9×10^−3^ μM of 5-FU, with or without 10^−4^ M of LF, 4–9.75×10^−4^ μM of DOXO. Cell proliferation was evaluated by MTT assay as previously described (11).

### Flow cytometric analysis of apoptosis

Annexin V binding was identified by flow cytometry using Annexin V-FITC staining, following the manufacturer’s instructions. Apoptotic cell death was also analyzed by propidium iodide (PI) detection systems (eBioscences, Vienna, Austria). Briefly, HaCaT cells were seeded in 6-well plates in a number of 15×10^4^ cells per well. After 24 h cells were treated with concentration inhibiting 50% of cell growth (IC_50_) of 5-FU, alone or in combination with 10^−4^ M of LF, and DOXO. After 24, 48 and 72 h of treatment cells were trypsinized, washed twice with PBS 1X and pellet were resuspended in 200 μl binding buffer 1X. Then we added 5 μl Annexin V-FITC to 195 μl cell suspension, mixed and incubated for 10 min at room temperature. Cells were washed in 200 μl binding buffer 1X and were resuspended in 190 μl binding buffer 1X, then we added 10 μl propidium iodide (20 μg/ml). The detection of viable cells, early apoptosis cells, late apoptosis cells and necrotic cells were performed by FACSAria™ (BD Bioscences). For each sample, 2×10^4^ events were acquired. Analysis was carried out by triplicate determination on at least three separate experiments.

### Flow cytometric analysis of oxidative stress

ROS generation was analyzed by flow cytometry using the ROS-sensitive dye dihydroethidium (DHE), a probe for measurement of super-oxide anion (O^2−^), as previously described (12). HaCaT cells were seeded in 6-well plates in a number of 15×10^4^ cells per well and were treated 24 h later with IC_50_ of 5-FU, alone or in combination with 10^−4^ M of LF, and DOXO. After 24, 48 and 72 h of treatment cells were incubated for 1 h at the end of treatment with 20 ng/ml dihydroethidium stock solution (2.5 mg/ml). We compared the effects induced by pharmacological agents treating HaCaT cells with 500 μM of H_2_O_2_, an inducer of superoxide anion formation, 2000 μM of N-acetylcysteine (NAC), a scavenger compound, and H_2_O_2_ in combination with NAC. At the time of processing the cells were trypsinized, washed twice with PBS 1X and the pellet was resuspended in 500 μl of PBS 1X. The dye accumulation was analyzed by BD FACSAria (BD Bioscences). For each sample, 2×10^4^ events were acquired. Analysis was carried out by triplicate determination on at least three separate experiments.

### Flow cytometric analysis of autophagy

Autophagy was analyzed by flow cytometry using monodansylcadaverine (MDC) staining. MDC is an auto-fluorescent agent used as selective marker for autophagic vacuoles (AVOs) and especially autolysosomes (13). Briefly, HaCaT cells were seeded in 6-well plates in a number of 15×10^4^ cells per well and were treated 24 h later with IC_50_ of 5-FU, alone or in combination with 10^−4^ M of LF and DOXO. After 24, 48 and 72 h of treatment cells were incubated with 50 μM of MDC in PBS 1X at 37°C for 15 min. After incubation, cells were washed twice in PBS 1X, trypsinized and the pellet was resuspended in 500 μl of PBS 1X. Samples were immediately analyzed by flow cytometry by BD FACSAria (BD Bioscences). For each sample, 2×10^4^ events were acquired. Analysis was carried out by triplicate determination on at least three separate experiments.

### Evaluation of thiobarbituric acid-reactive species (Tbars) levels

The levels of Tbars were analyzed using a spectrophotometric assay (14). For this purpose HaCaT cells were seeded at 1×10^6^ cells per well into 100 mm dishes. After 24, 48 and 72 h of treatment with IC_50_ of 5-FU, alone or in combination with 10^−4^ M of LF, and DOXO cells were washed twice in ice-cold PBS 1X, trypsinized and the pellets were collected. After cell lysis and the evaluation of protein concentration, samples were incubated with 0.5 ml of 20% acetic acid, pH 3.5, and 0.5 ml of 0.78% aqueous solution of thiobarbituric acid. After heating at 95°C for 45 min, the samples were centrifuged at 4000 rpm for 5 min. In the supernatant fractions Tbars were quantified by spectrophotometry at 532 nm (15). Results were expressed as Tbars μM/μg of serum protein. Analysis was carried out by triplicate determination on at least three separate experiments.

### Statistical analysis

All data are expressed as mean ± SD. Statistical analysis was performed by analysis of variance (ANOVA) with Neumann-Keul’s multiple comparison test or Kolmogorov-Smirnov where appropriate.

## Results

### Evaluation of cell growth inhibition of HaCaT cell line

**Figure 1:**
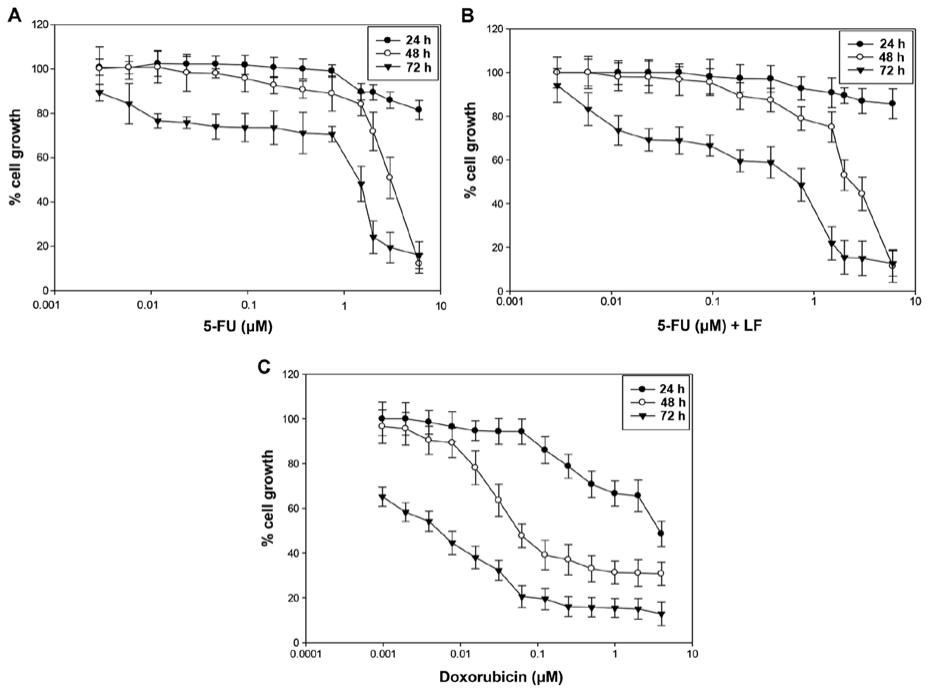
Effects of 5-fluorouracil (5-FU), 5-FU in combination with levofolene (LF) and doxorubicin (DOXO) on growth inhibition of HaCaT cell line. The cells were seeded and treated with different concentrations of 5-FU (A) or 5-FU+LF (B) or DOXO (C) for 24, 48 and 72 h and thereafter cell growth was evaluated by MTT assay as described in Materials and methods. The effects on cell growth were expressed as % of control. The experiments were performed three times. The bars represent means ± SD of three independent experiments.

In this study we evaluated the effects of 5-fluorouracil (5-FU) alone or in combination with levofolene (LF) and doxorubicin (DOXO) on the proliferation of the human keratinocyte (HaCaT) cells by MTT assay as reported in Materials and methods. We found that all the agents induced a time- and dose-dependent growth inhibition (Fig. 1A–C). The results are expressed as concentration inhibiting 50% of cell growth (IC_50_) after 72 h of treatment (Table I). The IC_50_ was reached with 1.4 μM of 5-FU (Fig. 1A), 0.7 μM of 5-FU in combination with 10^−4^ M of LF (Fig. 1B) and 0.005 μM of DOXO (Fig. 1C). These data suggested that human keratinocyte cells were more sensitive to the treatment with 5-FU in combination with LF compared to those treated with 5-FU alone, confirming that LF potentiated cytotoxic effects of 5-FU. However, HaCaT cells were more sensitive to the treatment with doxorubicin as IC_50_ was reached with a lower dose of drug after 72 h of treatment.

### Evaluation of apoptosis by flow cytometric analysis

**Figure 2:**
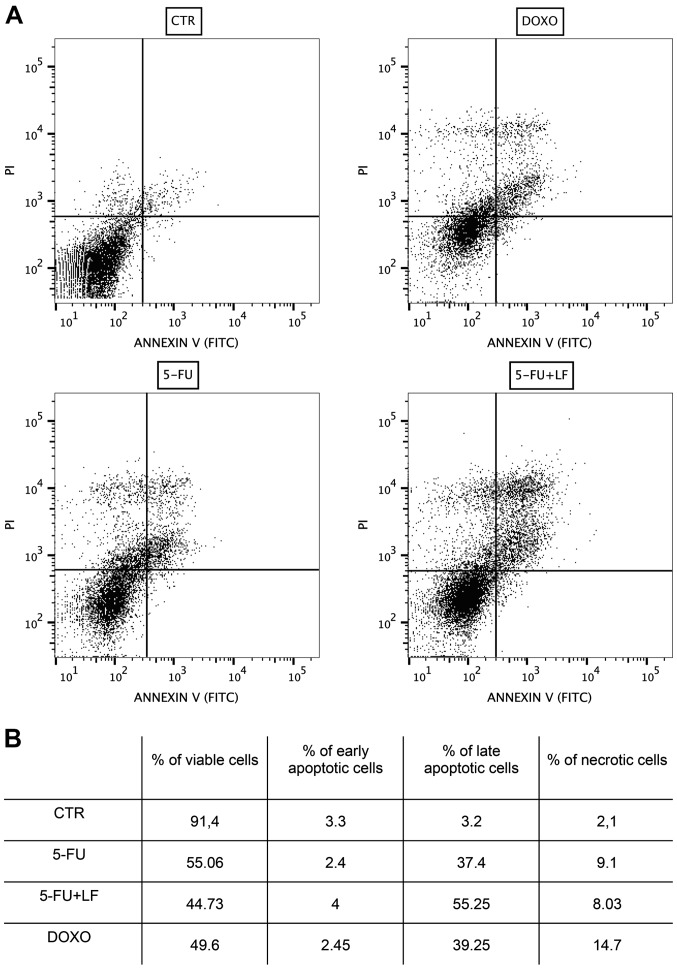
Evaluation of apoptosis by Annexin V/PI assay (flow cytometry) after 48 h of treatment with 5-fluorouracil (5-FU), 5-FU in combination with 10^−4^ M of levofolene and doxorubicin. (A) Flow cytometry dot plots. (B) Summary of data expressed as percentage of viable cells, early/late apoptotic cells and necrotic cells.

The effects of 5-FU, alone or in combination with LF and DOXO in inducing apoptosis or necrosis in HaCaT cell line were evaluated after treating cells for 48 h with IC_50_ of each compound, as reported in Materials and methods. We have found that 5-FU induced late apoptosis in ~37% of cells and necrosis in ~9% of cells while the combination with LF induced late apoptosis in ~55% of cells and necrosis in ~8% of cells. Noteworthy, we found that DOXO induced late apoptosis in only ~39% of cells and necrosis in ~15% of cells. The percentage of cells induced in early apoptosis by each pharmacological treatment was not significant (Fig. 2). These data suggested that after 48 h 5-FU damaged human keratinocytes principally through apoptosis and this effect was potentiated by the combination with LF while doxorubicin acted also through occurrence of necrosis.

### Evaluation of autophagy

**Figure 3:**
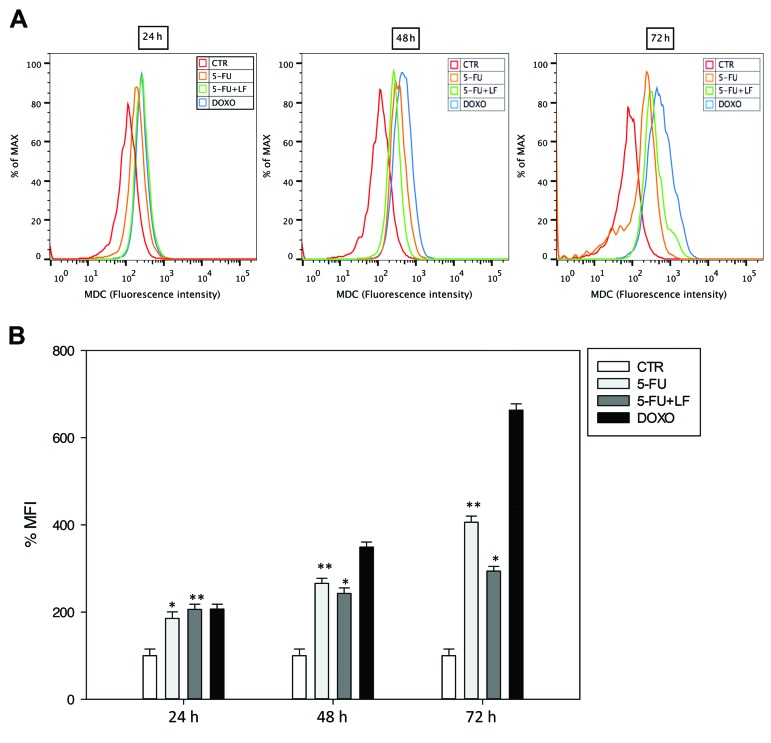
Evaluation of autophagy in HaCaT cells treated with 5-fluorouracil (5-FU), 5-FU in combination with levofolene (LF) and doxorubicin (DOXO) after 24-48-72 h. (A) Flow cytometry overlay of monodansylcadaverine (MDC) fluorescence intensity. (B) Histogram of MDC mean fluorescence intensity (% of control). The bars represent means ± SD of three independent experiments. Asterisks indicate significant difference between 5-FU-treated vs. 5-FU+LF-treated cells (^**^P<0.003) and untreated vs. 5-FU-treated cells (^*^P<0.05).

In this study we evaluated the effects of the pharmacological agents in inducing autophagy in HaCaT cell line as reported in Materials and methods. After 24 h, 5-FU induced an increase of ~185% of MFI while 5-FU in combination with LF induced ~206% of MFI such as DOXO (Fig. 3). Of note, after 48 and 72 h 5-FU increased the percentage of MFI in contrast to the combination with LF. 5-FU induced in HaCaT cells an increase of ~266 and 406% of MFI after 48 and 72 h, respectively, while the combination with LF induced an increase of ~242 and 294% of MFI after 48 and 72 h, respectively (Fig. 3). On the other hand, DOXO induced a time-dependent increase of autophagic vacuoles and the maximal effect was reached after 72 h with an increase of ~663% of MFI (Fig. 3). These results confirmed data obtained by apoptosis studies since 5-FU alone induced an increased cell death that lasted until the end of the treatment. LF potentiated autophagic effect of 5-FU only at 24 h but protected keratinocytes from cell death at longer exposure times.

### Evaluation of oxidative stress

**Figure 4:**
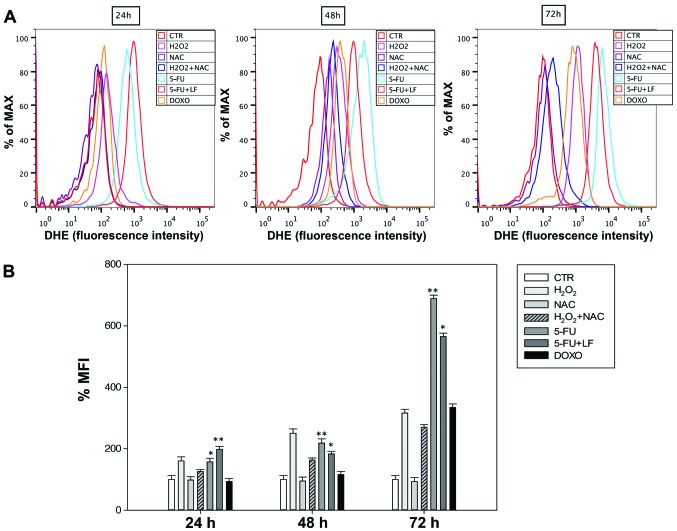
Evaluation of oxidative stress in HaCaT cells treated with 5-fluorouracil (5-FU), 5-FU in combination with 10^−4^ M levofolene (LF) and doxorubicin (DOXO) after 24-48-72 h. (A) Flow cytometry overlay of dihydroethidium (DHE) fluorescence intensity. (B) Histogram of DHE mean fluorescence intensity (% of control). The bars represent means ± SD of three independent experiments. Asterisks indicate significant difference between 5-FU-treated vs. 5-FU+LF-treated cells (^**^P<0.003) and untreated vs. 5-FU-treated cells (^*^P<0.05).

We evaluated the effects of 5-FU, 5-FU in combination with LF and DOXO on the accumulation of superoxide anions (O^2−^) in HaCaT cells as reported in Materials and methods. We observed after 24 h that 5-FU induced an increase of superoxide anions of ~157% of MFI against an increase of ~198% of MFI induced by the combination with LF (Fig. 4). However, after 48 and 72 h 5-FU in combination with LF induced an increase of O^2−^ levels significantly lower compared to that one induced by 5-FU alone. 5-FU induced in HaCaT cells an increase of superoxide anions of ~218 and 689% of MFI while the combination with LF induced an increase of superoxide anions of ~183 and 565% of MFI after 48 and 72 h, respectively (Fig. 4). On the other hand, we observed a time-dependent accumulation of ROS levels in HaCaT cells treated with doxorubicin but it was significantly lower compared to that one induced by other pharmacological treatments and the maximal increase of O^2−^ anions (~334% of MFI) was reached after 72 h (Fig. 4). NAC had no effect on the increase of O^2−^ levels and it acted as a scavenger in combination with H_2_O_2_ decreasing the accumulation of superoxide anions (Fig. 4). Therefore, these data suggested that LF in combination with 5-FU induced a protective effect on the formation of ROS in long-time exposure.

### Evaluation of the levels of thiobarbituric acid-reactive species (Tbars)

**Figure 5:**
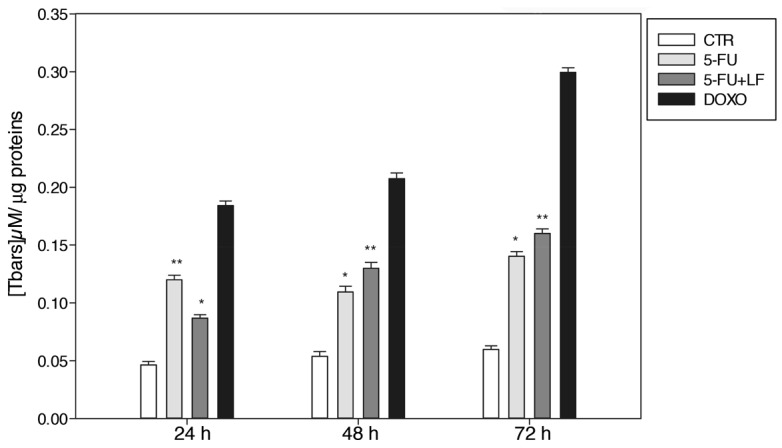
Effects of 5-fluorouracil (5-FU), 5-FU in combination with levofolene (LF) and doxorubicin (DOXO) on the level of Tbars in HaCaT cell line. The cells were seeded and treated with 5-FU or 5-FU+LF or DOXO for 24, 48 and 72 h. Thereafter, Tbars levels in the different experimental conditions were determined as described in Materials and methods. The bars represent means ± SD of three independent experiments.

In this study, we evaluated the effects of 5-FU, alone or in combination with LF, and DOXO on the modulation of the Tbars level in HaCaT cell line as reported in Materials and methods. After 24 h we found that 5-FU induced the formation of ~0.085 μM Tbars/μg of proteins, while 5-FU in combination with LF induced a lower formation of the Tbars level which was ~0.087 μM Tbars/μg of proteins (Fig. 5). Noteworthy, after 48 and 72 h of treatment the combination 5-FU/LF induced a greater amount of the Tbars species compared to that one induced by 5-FU alone. 5-FU in combination with LF induced the formation of 0.11 μM Tbars/μg of proteins and 0.15 μM Tbars/μg of proteins after 48 and 72 h, respectively, while 5-FU alone induced an amount of 0.10 μM Tbars/μg of proteins and 0.16 μM Tbars/μg of proteins after 48 and 72 h, respectively (Fig. 5). On the other hand, free doxorubicin caused a time-dependent accumulation of the Tbars level and the maximal was reached after 72 h with ~0.3 μM Tbars/μg of proteins (Fig. 5). These data suggest that the effects of the combination on the increase of lipid peroxidation were late when compared to those on intracellular O^2−^ increase suggesting that the decrease of O^2−^ levels at 48 and 72 h was, at least in part, due to the formation of reactive species with intracellular complex molecules such as lipids.

## Discussion

5-Fluorouracil (5-FU) has been known to cause hand-foot syndrome (HFS) since the first description by Lokich and Moore in 1984 (16). The continued prolonged exposure to 5-FU, provided by oral administration of capecitabine, leads to high incidence of HFS.

The clinical manifestation of HFS can be divided into four grades from slight dysesthesia to desquamation, blistering, and ulceration. The management of these side effects can also require the interruption of the therapy for the cancer disease depending upon the severity of HFS. In fact, the occurrence of HFS is never life-threatening, but can develop into a debilitating condition that may severely interfere with the quality of life (17).

The pathophysiological mechanisms of HFS are an active area of investigation. The involved factors could be the following: i) rapid cell division rate of palm and sole keratinocytes, ii) gravitational forces, iii) peculiar vascular anatomy of these areas, iv) temperature gradients in the distal extremities, v) increased levels of thymidine phosphorylase in keratinocytes. On the basis of these considerations, 5-FU can have increased cytotoxic effects in these areas if compared with other body skin sites (6,18). Moreover, the oral 5-FU derivative capecitabine can be preferentially eliminated by eccrine glands, resulting in increased excretion in palms and soles that have higher number of these glands. Histologic features of HFS are non-specific. They include vacuolar degeneration of the basal cell layer, mild spongiosis, keratinocyte necrosis, papillary dermal edema, lymphohistiocytic infiltrates and partial separation of the epidermis from the dermis (19).

In case of direct topic contact with 5-FU, the histologic alterations involve keratinocytes in the lower third of the epidermis. The main intracellular identified abnormalities described are dilatation of endoplasmatic reticulum and Golgi complex, membrane-limited perinuclear vacuoles and degeneration of mitochondria (6,18). Concerning the management of chemotherapy-induced HFS, discontinuation of the drug, or dose modifications, are the only available recommendations. Some measures suggested to control HFS symptoms include cold compresses, application of emollients and to avoid excessive pressure to the skin and extreme temperatures. Topical corticosteroids or dimethylsulfoxide have been also used, but with no definitive results. Oral or topical pyridoxine (vitamin B6) and topic use of vitamin K have been successfully used in some instances (20). However, the efficacy of these measures has not been reported in controlled trials and emphasis is placed on the management of symptoms as they manifest and progress (6,21). Therefore, the identification of the causes that lead to the development of the HSF is a key element for therapy optimization.

It is also important to underline that the appearance of toxic effects is also possible in exposed workers, as well as in patients undergoing chemotherapy. In 5 Japanese hospitals, 5-FU had the highest concentration detected in working table, on floor and in air-conditioner filters (43±44 ng/m^2^; 5.2±4.2 ng/m^2^ and 4600 ng, respectively) outside the laminar flow hood, where operators do not use protective gloves (22). It has also to be considered that 5-FU is used often in combination with LF that has largely demonstrated to potentiate its anticancer effects (10,23). To our knowledge, studies on the exposure of workers and patients to both 5-FU and LF have not yet been reported and, similarly, the interaction of the two drugs on normal human keratinocytes has not yet been reported.

In the present study, we show that 5-FU can induce keratinocyte growth inhibition at concentrations that are close to those reported in air-conditioner filters of hospitals and that can be, therefore, potentially detrimental for both patients and workers that are continuously exposed to this drug for long periods of time. Moreover, the combination of 5-FU with LF potentiates the anti-proliferative effects of the anticancer drug on keratinocytes suggesting that the co-exposure to both agents can increase not only the therapeutic action of 5-FU but also its detrimental effects on skin. Of note, the growth inhibition induced by 5-FU appeared to be induced by the occurrence of apoptosis paralleled by markers of an increased oxidative stress as both increased superanion levels and higher membrane lipid peroxidation. These results suggest an activation of a mitochondria-dependent apoptotic mechanism since the increase of intracellular ROS is at the basis of mitochondria membrane potential transition and the consequent release of cytochrome c (24). In our experimental model, a time-dependent increased autophagic vacuole accumulation was observed in keratinocytes treated with 5-FU as a significant increase of the MDC labeling (marker of late autophagy vacuoles) was recorded. However, the synergism of 5-FU with LF on apoptotic occurrence was not paralleled by a similar interactive increase in autophagic vacuoles at 72 h. These results suggest that the autophagy observed in 5-FU-treated keratinocytes could be a protective escape mechanism from apoptotic cell death. Therefore, cells exposed to LF have antagonizing effects on autophagy thus increasing the apoptotic cell death. In this light, it was recently reported that the antidepressant agent and ubiquitin ligase ITCH1 inhibitor desmethylclomipramine had the ability to block the autophagic flux that was paralleled by a paradoxical increase of biochemical markers of autophagic vacuoles (AVOs) (25). Moreover, the treatment of cancer cells with cytotoxic drugs together with desmethylclomipramine potentiated apoptosis and caused an additional accumulation of autophagic vacuoles suggesting that the block of autophagic flux induced by desmethylclomipramine can favor apoptosis caused by cytotoxic agents (26).

Differential effects on reactive oxygen species (ROS) elevation in cells treated with 5-FU alone or the combination between 5-FU and LF were also observed. 5-FU induced a time-dependent increase of both O^2−^ and lipid peroxidation while the combination of 5-FU and LF caused a stronger intracellular O^2−^ increase only at 24 h while at 48 and 72 h its effect was lower when compared with that of 5-FU alone. On the other hand, the addition of LF to 5-FU caused a stronger increase of lipid peroxidation at 48 and 72 h, but its effects were significantly lower at 24 h. Therefore, it can be hypothesized that the combination of 5-FU and LF can elicit apoptosis through the triggering of cell death mechanisms different from oxidative stress-dependent activation of apoptosis.

Moreover, the late increase of lipid peroxidation levels induced by the combination compared to 5-FU alone and the early increase of O^2−^ levels suggests that superanions are consumed during the oxidative reaction with intracellular complex molecules such as lipids. We have also previously reported that the same combination has potent apoptotic effects on cardiomyocytes (27). In those experimental conditions, the synergistic effect of the two agents on apoptosis was paralleled by a strong oxidative stress occurrence and by mitochondrial membrane potential transition (28). On these bases, it can be suggested that the change of the experimental model may have relevance on the mechanisms of interaction between 5-FU and LF.

In conclusion, our results show that LF pharmacologically interact with 5-FU potentiating the growth inhibition and apoptosis of human keratinocytes, and that these effects occur together with antagonistic effects on autophagy suggesting the interruption of an anti-apoptotic escape mechanism. These effects are paralleled by a dynamic induction of oxidative stress in human keratinocytes. These data strongly support the design of studies aimed to investigate the exposure of patients and workers to 5-FU and LF.

## Figures and Tables

**Table I:** Concentrations inhibiting 50% of cell growth (IC_50_) in HaCaT cells after 72 h of treatment with 5-FU, 5-FU in combination with 10^−4^ M of LF and doxorubicin.^a^

Compounds	IC_50_ ± SD
5-FU	1.4±0.04 μM
5-FU+LF	0.7±0.01 μM
DOXO	0.005±0.03 μM

a Data are expressed as mean ± SD.

5-FU, 5-fluorouracil; LF, levofolene; DOXO, doxorubicin.
